# Breast cancer susceptibility gene 1 (*BRCA1*) predict clinical outcome in platinum- and toxal-based chemotherapy in non-small-cell lung cancer (NSCLC) patients: a system review and meta-analysis

**DOI:** 10.1186/1756-9966-32-15

**Published:** 2013-03-15

**Authors:** Yanlong Yang, Yuanliang Xie, Lei Xian

**Affiliations:** 1 Department of Cardiothoracic Surgery, the First Affiliated Hospital of Guangxi Medical University, Nanning, Guangxi Zhuang Autonomous Region, 530021, China; 2 Department of Urology, the First Affiliated Hospital of Guangxi Medical University, Nanning, Guangxi Zhuang Autonomous Region, 530021, China

**Keywords:** BRCA1, NSCLC, Platinum, Toxal, Meta-analysis

## Abstract

The recent studies have evaluated the relationship between *BRCA1* expression and clinical outcome of chemotherapy (mainly focused on platinum-based and toxal-based treatment) in NSCLC patients, but the results were inconclusive and controversial. Our aim of this study was to evaluate this association by literature based system review and meta-analysis.

PubMed, EMBASE and the China National Knowledge Infrastructure (CNKI) databases were used to retrieve the relevant articles. The interested outcome included objective response rate (ORR), overall survival (OS) and event-free survival (EFS). The pooled odds ratio (OR) or hazard ratio (HR) with 95% confidence interval (CI) ware estimated.

After specific inclusion and exclusion criteria, 23 studies fulfilled the criteria and were included in our analysis. In 17 platinum-based studies, low/negative *BRCA1* was in favor of better ORR (OR = 1.70, 95%CI = 1.32-2.18), longer OS and EFS (HR = 1.58, 95%CI = 1.27-1.97, and HR = 1.60, 95%CI = 1.07-2.39 for OS and EFS, respectively). In 4 toxal-based chemotherapy studies, the patients with high/positive *BRCA1* had better ORR (OR = 0.41, 95%CI = 0.26-0.64), OS and EFS were not evaluated as the insufficient data available.

Overall, *BRCA1* might be a useful biomarker to predict clinical outcome for personal chemotherapy in NSCLC patients in the future.

## Introduction

Lung cancer is now the most commonly diagnosed cancer and the leading cause of cancer death worldwide [1]. In USA, 412,230 cases had lung cancer history and the new cases estimated in 2012 were 226,160. Most of lung cancers (56%) are diagnosed at an advanced stage as the typically asymptomatic in early stage. Lung cancer is classified into primarily two subgroups: small-cell lung cancer (SCLC) and non-small-cell lung cancer (NSCLC), and the later accounts for approximately 85% of all lung cancers. Although the notable progress has been made in lung therapy, this disease is still associated with a poor prognosis and has few effective treatment options. The overall 5-year survival rate for NSCLC is 17.1% [2].

The chemotherapy efficacy is varied from different individual, even in patients with similar clinical and pathologic features, the outcome varies: some complete released, some are stable or even progression. So some authors consider NSCLC as a heterogeneous disease [3]. The genetic factor may contribute greater to this diverse efficacy, the SNPs or gene expression products (mRNA and protein) could be special biomarkers. As the development of biochemistry, some notable biomarkers such as EGFR, VEGF, were identified to predict lung cancer treatment outcome, breast cancer susceptibility gene 1 (*BRCA1*) has emerged as one of the most appealing genetic markers among them.

*BRCA1* located in chromosome 17q21, and was identified as a breast and ovarian cancer susceptibility gene. *BRCA1* germline mutations have been correlated to the increasing risk of developing breast and ovarian cancer [4,5]. Recent studies have shown that the protein encoded by this gene is a nuclear phosphoprotein and has multiple roles not only in DNA damage repair but also in cell cycle checkpoint or cell death machinery [6,7]. A greater sensitivity to cisplatin with decreased *BRCA1* mRNA expression and a greater resistance to the paclitaxel with increased *BRCA1* mRNA expression was observed in breast cancer cell lines [7,8]. Also in tumour cells isolated from malignant effusions of NSCLC and gastric cancer patients, the same effect was observed [9].

Followed by *in vitro* studies, clinical studies explored this relationship. Taron *et al.*[10] firstly examined the potential role of *BRCA1* mRNA expression in predicting differential chemotherapy sensitivity in NSCLC, and found the patients with high *BRCA1* had poor outcome while those with low had better outcome. Followed by Taron, a series of studies evaluated the relationship between *BRCA1* level and chemotherapy outcome. The chemotherapy regimens were mainly focused on platinum-based and toxal-based treatment. However, the results were inconclusive due to the limited sample size and the limited statistics power. Current study provided a comprehensive assessment on the association between *BRCA1* level and the platinum- and toxal-based chemotherapy in NSCLC using meta-analysis.

## Materials and methods

### Literature search

Relevant studies were searched in PubMed, EMBASE and China National Knowledge Infrastructure (CNKI) databases using the following terms: “*BRCA1* or Breast cancer susceptibility gene 1 or Breast cancer 1” and “NSCLC or non-small-cell lung cancer”. The last research time was December 10, 2012.

### Inclusion criteria

The following criteria were used to select publications: (1) studies published in English and Chinese regardless of publication time; (2) reviews, animal or cell line studies should be excluded; (3) the NSCLC patients should be pathologically confirmed; (4) *BRCA1* expression should be detected by immunohistochemistry (IHC) or reverse-transcriptase polymerase chain reaction (RT-PCR); (5) the studies should provided the clinical outcomes such as objective response rate (ORR) to chemotherapy, overall survival (OS) or event-free survival (EFS) with HR and 95%CI, EFS was classified as progression-free survival (PFS), disease-free survival (DFS) and time to progression (TTP).

### Data collection

Publication characteristics details such as first author’s name, publication year, patients’ original country, sample type, detection method of *BRCA1*, sample size, middle/mean age of study sample, disease stage were collected for each eligible publication. End points of interest were objective response rate (ORR), overall survival (OS), and event-free survival (EFS).

### Statistical analysis

To estimated ORR, the patients were divided into responders and non-responders. The responders were defined as complete response (CR) and partial response (PR) and the non-responders including stable disease (SD) and progressive disease (PD). The pooled odds ratio (OR) and its 95% confidence intervals (CIs) were calculated by the methods proposed by Mantel and Haenszel [11], or by DerSimonian R and Laird N [12]. For time-to-event data-OS and EFS, the hazard ratios (HRs) and associated 95% confidence interval (CI) were estimated using the methods reported by Parmar [13].

The between study heterogeneity was determined by Q test and *I*^2^ metric (*I*^2^ = 0–25%: no heterogeneity; *I*^2^ = 25–50%: moderate heterogeneity; *I*^2^ = 50–75%: large heterogeneity; *I*^2^ = 75–100%: extreme heterogeneity) [14]. The fixed-effect model was applied in the initial analysis, and if the significant heterogeneity existed, then the confirmed random-effect model was used. Begg’s test was used to evaluate the publication bias. P < 0.05 indicated significant publication bias [15]. All *P* value was two-tailed, and STATA version 11.1 (Stata Corporation, USA) was used to perform the most of data analysis.

## Results

### Eligible studies

**Figure 1:**
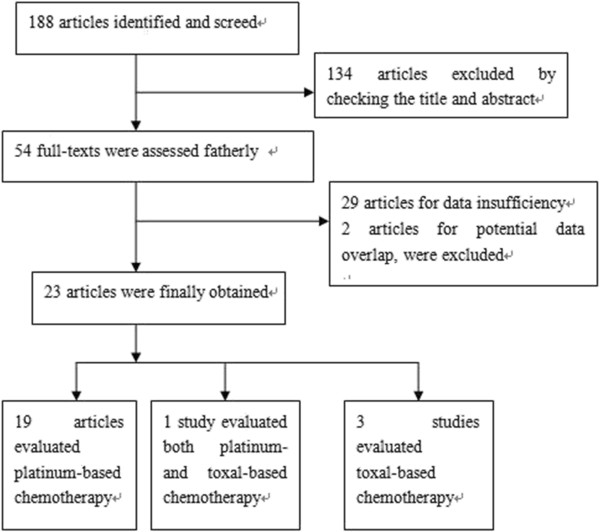
The flow chart of study selection and exclusion.

188 potentially relevant studies were identified through the search strategy. After checking the title and abstract, 134 studies excluded because it was very clear that their design didn’t meet our inclusion criteria. Then the full texts of 54 articles were carefully screened, 29 studies were excluded as data insufficiency that we could not extract the data for analysis, 2 studies were excluded for potential data overlap as the same institute conducted the research and their patients recruitment time may exist overlap. Finally, a total of 23 studies were eligible for the final analysis. Among them, 19 studies estimated the relationship between *BRCA1* and platinum-based chemotherapy outcome [10,16-33], 3 were toxal-based [34-37]. Additional one studies evaluated the toxal-based in fist-line chemotherapy and a part of patients received platinum-based treatment [36]. The study selection process was showed in Figure 1.

### Study characteristics

Our meta-analysis composed 23 studies [10,16-37] including 2606 NSCLC patients. The sample size variant from 34 to 769, 17 studies were about East-Asian population [16-25,27,28,30,32-34,37], 5 studies were about Caucasian [10,26,29,35,36] and 1 studies may contain different races as the samples were from the prospective randomized clinical trial International Adjuvant Lung Trial (IALT) [31]. In this multi-center study, the author’s intention was to investigate the predictive and prognostic role of *BRCA1* level in squamous cell carcinoma (SCC) and adenocarcinoma (ADC), so both OS and EFS were conducted in 2 groups (SCC and ADC). Table 1 summarized the main characteristics of included studies.

**Table 1:** **Characteristics of eligible studies evaluating *BRCA1 *level and clinical outcome**

**Study (year)**	**Source of study**	**No. of patients**	**median age**	***BRCA1 *detection**	**Disease stage**	**Chemotherapy**	**Clinical outcome**
Taron,2004 [10]	Spanish	60	NR	RT-PCR	llb,lll	GP	ORR,OS
Ota,2009 [16]	Japan	156	62	IHC	IV	NP,DC,PI,GP,paclitaxel/carboplatin	ORR,
Shang,2009 [17]	China	60	54	IHC	llllll	platinum-based	ORR
Yang,2009 [18]	China	75	57	RT-PCR	lllB, IV	NP,TP	ORR,OS
Shan,2009 [19]	China	81	62	IHC	lllB, IV	NP,GP,TP	ORR
Wang,2010 [20]	China	34	61	RT-PCR	lllB, IV	GP	ORR
Lu,2010 [21]	China	65	62.4	IHC	lllB, IV	GP	ORR
Mo,2011 [22]	China	80	50	IHC	lll, IV	GP,NP,TP	ORR
Gao,2011 [23]	China	122	60	IHC	lllB, IV	platinum-based	ORR
Wan,2011 [24]	China	87	58	IHC	lllB, IV	TP	ORR
Zhang,2011 [25]	China	136	61	IHC	lll, IV	GP,NP,TP	ORR
Chen,2011 [33]	China	152	NR	IHC	lllB, IV	GP,NP,TP	ORR
Joerger,2011 [26]	Netherlands	42	59.3	IHC	lllB, IV	GP	ORR,OS,PFS
Fujii,2011 [27]	Japan	35	58	IHC	lll	neoadjuvant chemotherapy and chemoradiotherapy(PI,DC)	ORR,OS
Gu,2012 [28]	China	50	NR	IHC	llllll	neoadjuvant chemotherapy(NP,GP)	ORR
Papadaki,2012 [29]	Greece	100	63	RT-PCR	IV	2nd line PI,Cisplatin,Cisplatin + pemetrexed	ORR,OS,PFS
Zeng,2010 [30]	China	63	64	IHC	llllll	NP,GP,EP	OS
Pierceall,2011 [31]	Multi-center	769	NR	IHC	llllll	platinum-based,no treatment	OS,DFS
Leng,2012 [32]	China	85	57	RT-PCR	llllll,IV	GP,NP,TP	OS,DFS
Boukovinas,2008 [36]	Greece	96	60	RT-PCR	lllB, IV	1st line DG,2nd line platinum-based	ORR,OS,TTP
Su,2011 [34]	China	63	60	RT-PCR	lllB, IV	toxal-based	OS,
Papadaki,2011 [35]	Greece	131	60	RT-PCR	lllB, IV	DG,DC	ORR,OS,PFS
Zhou,2012 [37]	China	64	58	IHC	lll, IV	toxal-based	ORR

Note: RT-PCR: real-time reverse transcriptase polymerase chain reaction, IHC: immunohistochemistry, GP: gemcitabine/platinum, NP: vinblastine/platinum, DC: docetaxel/cisplatin, PI: platinum/irinotecan, TP: toxal/platinum, NR not reported, PFS: progression-free survival, DFS: disease-free survival, TTP: time to progression.

### *BRCA1* level and the clinical outcome of chemotherapy

**Figure 2:**
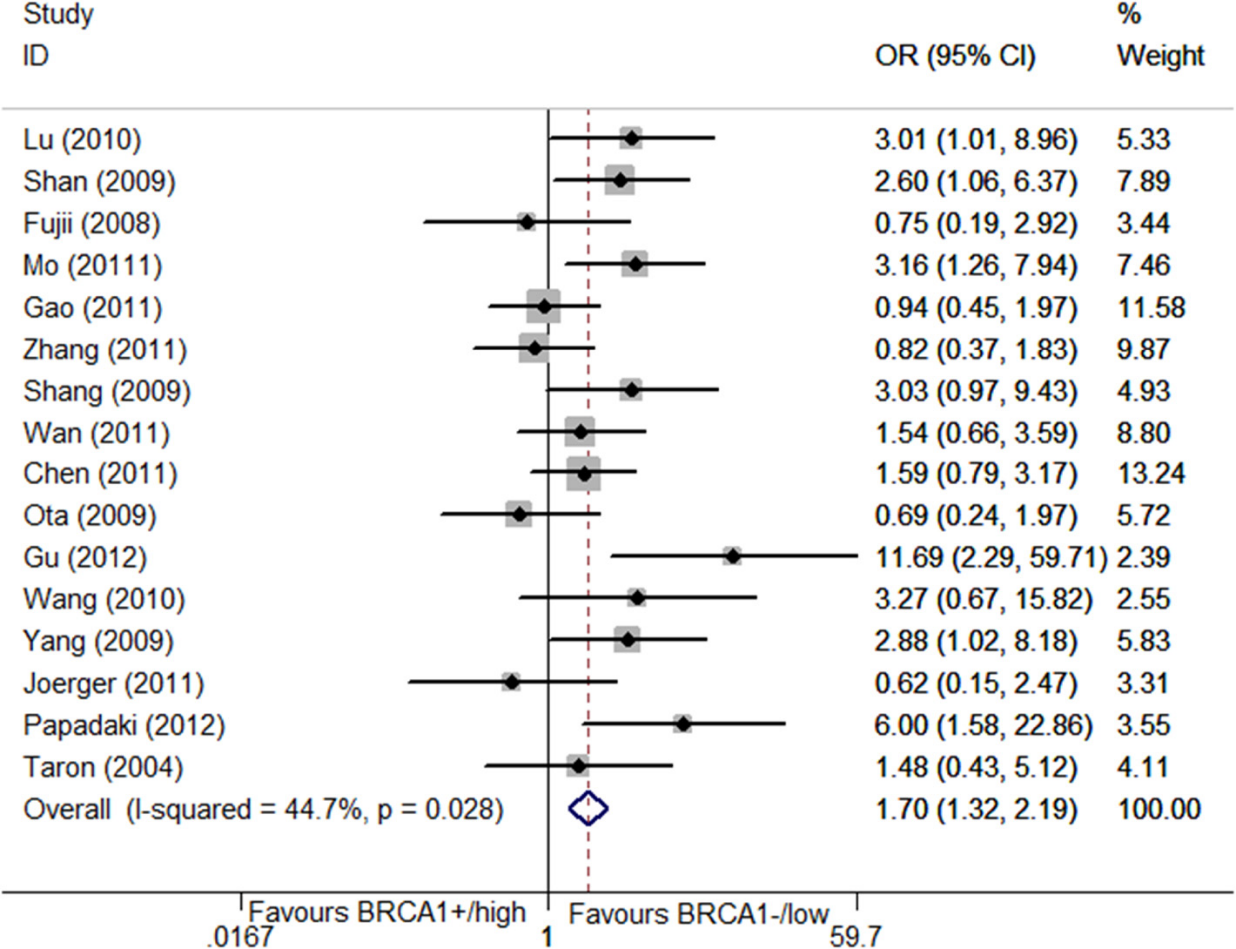
Forest plot for the association between BRCA1 level and objective response rate (ORR) in platinum-based treatment.

**Figure 3:**
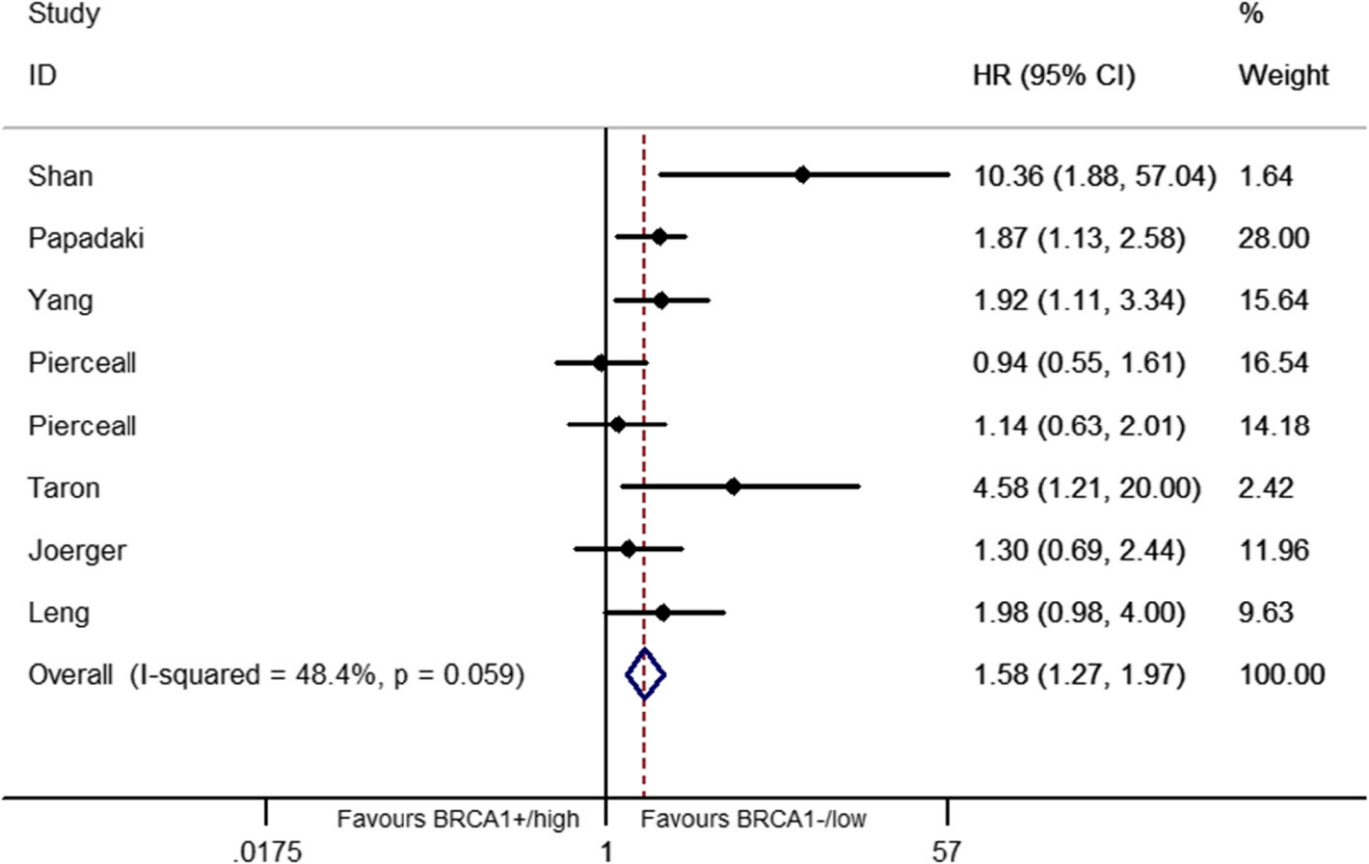
Forest plot for the association between BRCA1 level and overall survival (OS) in platinum-based treatment.

**Figure 4:**
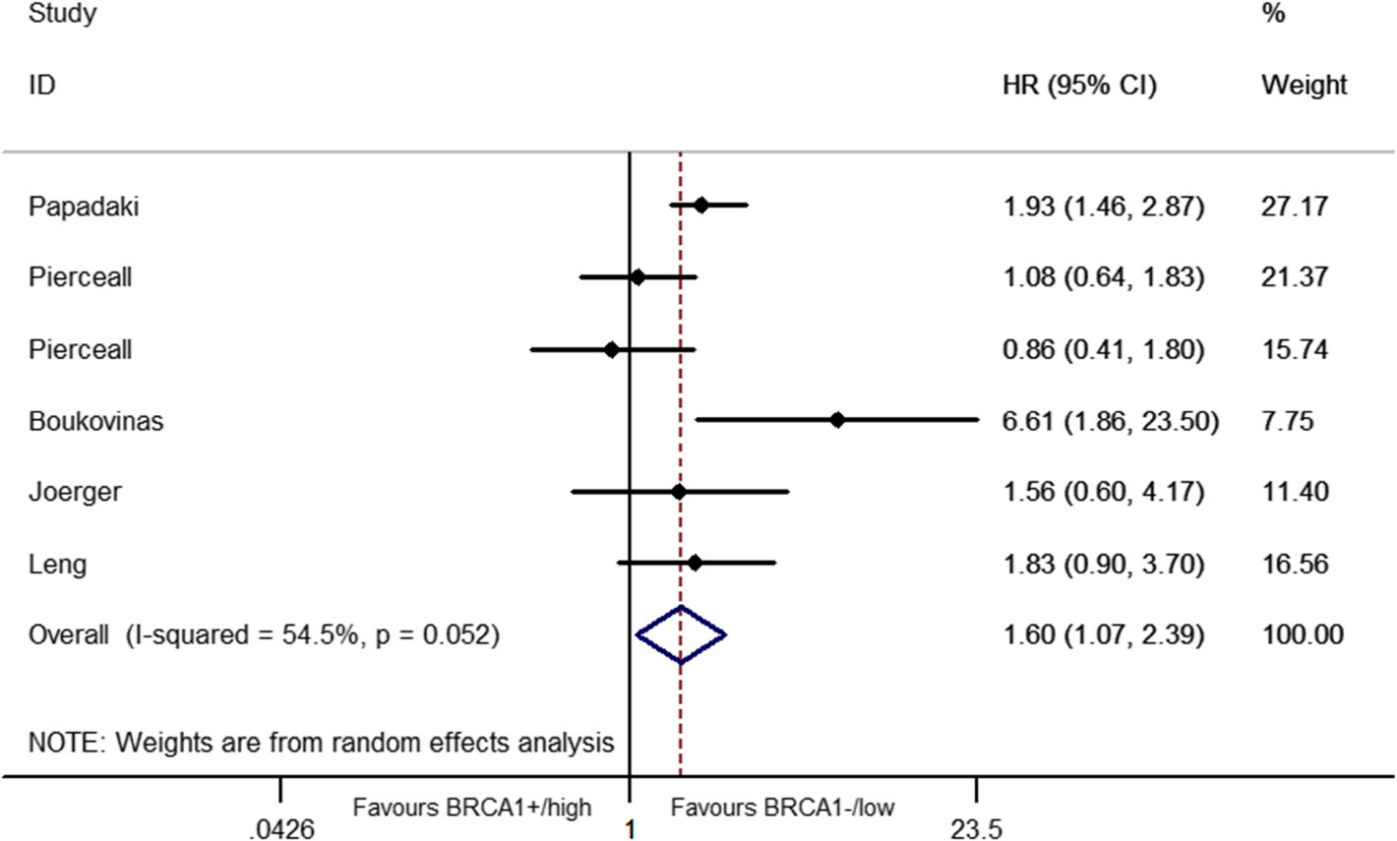
Forest plot for the association between BRCA1 level and event-free survival (EFS) in platinum-based treatment.

**Figure 5:**
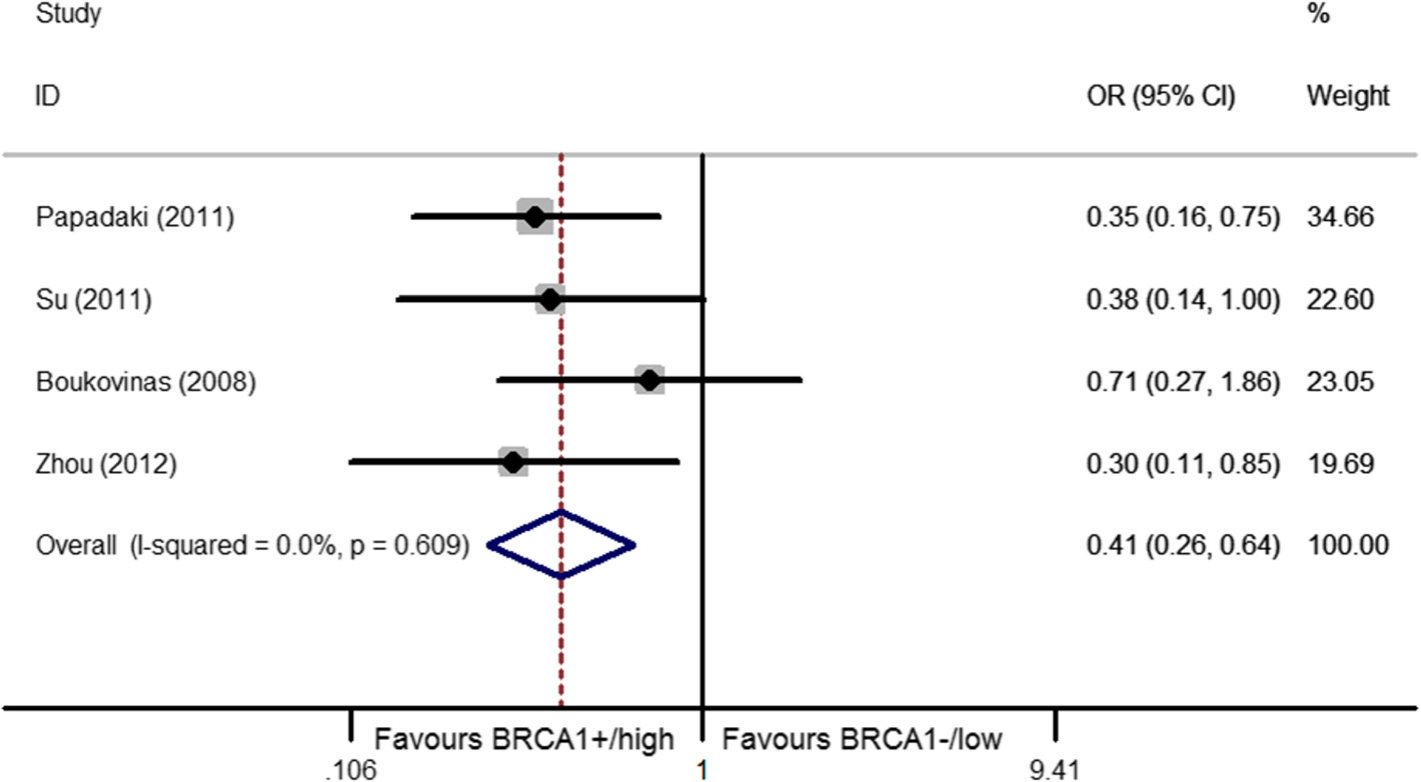
Forest plot for the association between BRCA1 level and objective response rate (ORR) in toxal-based treatment.

The relationship between *BRCA1* level and the clinical outcome was presented in Table 2 and Figures 2, 3, 4, 5.

1. Platinum-based chemotherapy

16 studies [10,16-29,33] composed 1330 patients reported the data on ORR. Low/negative *BRCA1* expression was observed in 591 patients (account for 44.4%) and the high/positive expression was in 739 patients (55.6%). It seemed that patients bearing low/negative *BRCA*1 had a higher ORR to platinum-based chemotherapy than those bearing high/positive *BRCA1* level (48.9% vs 38.1%, OR = 1.70, 95%CI = 1.32-2.18, *I*^2^ = 44.7%, *P* = 0.03 for heterogeneity) (Figure 2). No publication bias was observed (*P* = 0.15).

In subgroup analysis based on *BRCA1* detection method, there were 13 IHC studies (1066 patients) [16,17,19,21-28,33] and 4 RT-PCR studies (264 patients) [10,18,20,29], the distribution of low/negative *BRCA1* was similarity(IHC vs RT-PCR: 44.5% vs 44.3%). Both of them found the significant association (for IHC studies, 50.7% vs 39.0%, OR = 1.54, 95%CI = 1.17-2.00, *I*^2^ = 44.8%, *P* = 0.03 for heterogeneity; for RT-PCR studies, 43.7% vs 25.0%, OR = 2.91, 95%CI = 1.55-3.83, *I*^2^ = 0.0%, *P* = 0.52 for heterogeneity), When we stratified studies according to their origin, 13 studies were conducted in East-Asian [16-25,27,28,33] and only 3 were Caucasian [10,26,29]. The low/negative *BRCA1* level distribution in Caucasian was lower than East-Asian (38.6% vs 45.4%).The significant association was found in East-Asian population rather than Caucasian: for East-Asian, 51.0% vs 36.0%, OR = 1.68, 95%CI = 1.30-2.19, *I*^2^ = 39.9%, *P* = 0.04 for heterogeneity; for Caucasian, 39.8% vs 33.4%, OR = 1.77, 95%CI = 0.50-6.28, *I*^2^ = 63.6%, *P* = 0.06 for heterogeneity. However, the relationship between *BRCA1* level and ORR in Caucasian population could not be determined as the sample size was not large enough.

7 studies consisted of 3 East-Asian [18,30,32] and 4 Caucasian [10,26,29,31] including 733 patients were used to analyzed the OS. The significant association between *BRCA1* expression and OS in platinum-based treatment was detected. Patients bearing low/negative *BRCA1* was more likely to have longer survival time. (HR = 1.58, 95%CI = 1.27-1.97, *I*^2^ = 48.4%, *P* = 0.03 for heterogeneity) (Figure 3), no publication bias was observed (*P* = 0.13).

EFS data were available for 5 studies [26,29,31,32,36] with 599 patients (3 were PFS [26,29,32], one was DFS [31] and the other one was TTP [36]),only one study was about East-Asian[32]. It seemed that patients with low/negative *BRCA1* had longer EFS than those with high level, even there was no publication bias, but heterogeneity existed between studies. (HR = 1.60, 95%CI = 1.07-2.39) (*I*^2^ = 54.5%, *P* = 0.02 for heterogeneity) (Figure 4).

2. Taxol-based chemotherapy

Since only 2 studies [35,36] presented the sufficient data of OS and EFS that ensured us to conducted meta-analysis. We didn’t evaluate the relationship between *BRCA1* expression and OS/EFS. In ORR analysis, we applied 4 eligible studies (2 East-Asian and 2 Caucasian) [34-37] in our meta-analysis. A total of 375 patients were included in this comparison. Among 375 patients, 155 patients (account for 41.3% of total) bared low/negative *BRCA1* and the remaining 220 patients (account for 58.7% of total) bared high/positive *BRCA1*. An interesting conclusion was found: opposite to platinum-based treatment, NSCLC patients bearing high/positive *BRCA1* were more likely to respond to toxal-based treatment when compared with those bearing the low/negative (low/negative vs high/positive: 26.0% vs 46.1%, OR = 0.41, 95%CI = 0.27-0.64, I^2^ = 0.0%, *P* = 0.61 for heterogeneity) (Figure 5). No publication bias existed (*P* = 0.84).

**Table 2:** **The summary meta-analysis results of association between *BRCA1 *level with objective response rate (ORR), overall survival (OS) and event-free survival (EFS) in platinum- and toxal-based treatment**

**Comparisons**	**No of studies (patients)**	**Percentage of low/negative *BRCA1 *(%)**	**ORR: low/negative vs high/postive (%)**	**Overall OR/HR (95% CI) fixed and random**	**Heterogeneity test**	***P *for publication bias**
Platinum-based						
ORR overall	16(1330)	44.4	48.9 vs 38.1	1.70 (1.32, 2.18), 1.80(1.26,2.55)	*I*^2^ = 44.7%,P = 0.03	0.15
Method						
IHC	13(1066)	44.5	50.7 vs 39.0	1.54(1.17,2.00), 1.59(1.07,2.36)	*I*^2^ = 44.8%,P = 0.03	0.41
RT-PCR	4(264)	44.3	43.7 vs 25	2.91 (1.55, 3.83), 2.91(1.55,5.47)	*I*^2^ = 0.0%, P = 0.52	0.76
Origin						
East-Asian	14(1133)	45.4	51.0 vs 36.0	1.68(1.30,2.19), 1.79(1.24,2.60)	*I*^2^ = 39.9%,P = 0.04	0.10
Caucasian	3(197)	38.6	39.8 vs 33.4	1.79 (0.84, 3.83), 1.77(0.50,6.28)	*I*^2^ = 63.6%,P = 0.06	0.90
OS	8(733)	-	-	1.58(1.27,1.97), 1.65(1.19,2.89)	*I*^2^ = 48.4%,P = 0.03	0.13
EFS	6(599)	-	-	1.62(1.28,2.05), 1.60(1.07,2.39)	*I*^2^ = 54.5%,P = 0.02	0.88
Toxal-based						
ORR overall	4(376)	41.3	26.0 vs 46.1	0.41(0.26,0.64), 0.41(0.27,0.64)	*I*^2^ = 0.0%, P = 0.61	0.84

## Discussion

Although the relationship between *BRCA1* expression and chemotherapy outcomes of NSCLC has been investigated by previous studies, the results were inconsistent and some were even conflicting. So a systematic review and meta-analysis based on the published literature was necessary to give further insights on this conflicting issue. Our meta-analysis showed that for platinum-based chemotherapy, low/negative *BRCA*1 expression were associated with not only better ORR, but also longer OS and EFS, but for toxal-based chemotherapy, high/positive *BRCA1* was associated with better ORR.

Platinum agents can bind to DNA and form complexes thus inducing intra- and inter-strand DNA, as well as DNA-protein cross-links and results in cell growth inhibition and apoptosis. As one of ant-tubulin agents, taxol inhibits cell division by enhancing formation and stabilization of microtubules and disrupts the mitotic spindle assembly, and a surveillance mechanism known as the spindle checkpoint at the metaphase-anaphase transition have been activated. It leads to the abrogation of the spindle checkpoint, results in unequal segregation of chromosomes and aneuploidy [38].

Like excision repair cross complementing 1 (*ERCC1*), xeroderma pigmentosum group D and G (*XPD*, *XPG*), *BRCA1* belongs to nucleotide excision repair (NER) system [39], which has been reported to be the mayor repair system that reduced platinum-induced DNA damage. *BRCA1* involves in homologous recombination, nonhomologous end joining, mismatch repair and other effects though its interaction with other DNA repair gene such as *ATM*, *ATR*, *RAD51*, *RAD50*, *MRE11*, *NBS1*. *BRCA2* and so on [7]. The reason that high/positive *BRCA1* could predict the good response to taxol is still not clear, 3 mechanisms had been proposed in explained this issue: (1) trigger cell cycle arrest in G2/M phase, (2) enhance apoptosis through a pathway involving H-Ras, MEKK4, JNK, and activation of caspases 8 and 9, (3) participate in spindle assembly checkpoint signaling [6,40].

*BRCA1* gene showed an interesting outcome in NSCLC chemotherapy. Several cell studies and our meta-analysis based on clinical trials demonstrated low/negative *BRCA1* expression could benefit from platinum-based chemotherapy; in contrast, the high level of BRCA1 expression was in favor of toxal contained agents. This may confuse us, how could we determine chemotherapy choice properly? Rosell customized treated 84 patients based on their *BRCA1* expression: low, cisplatin plus gemcitabine (GP); intermediate, cisplatin plus docetaxel (DC); high, docetaxel alone. The median survival (MS) and 2-year survival of low *BRCA1* patients received GP regime was 11 month and 41.2%, which seem to be favorable with the traditional randomized trial treated with GP or pemetrexed plus cisplatin. The MS of high *BRCA1* patients received single-agent docetaxel was 11 month and had no detrimental effect when compared with a large phase III trial in patients treated with DC [41]. If this hypothesis is validated, the NSCLC patients with high *BRCA1* should receive taxol based and non-platinum-contained adjuvant chemotherapy, which would be more economic, efficacy and less toxic effect for patients. However, more multi-center prospective clinical trials should be conducted to confirm this hypothesis.

Since *BRCA1* mRNA and protein level was associated with treatment efficacy, why other biomarkers such as SNPs in this gene could be a choice? But in another hand, it seems that gene expression level provides direct evidence and SNPs provide indirect evidence as it is usually gene product especially protein rather than gene itself play an import role in biochemical activity. Although SNPs are important gene variant that affect the protein expression, but many factors involve in protein synthesis. We found that studies evaluated the SNPs in *BRCA1* gene and the clinical outcome was limited. Su [42] found that *BRCA1* S1613G was associated with platinum-based chemotherapy efficacy in objective response rate. In a large trail consisted of 300 NSCLC patients at stages III and IV, AACC haplotype but not single S1613G in *BRCA1* was associated with poor overall survival (hazard ratio = 2.097; 95%CI, 1.339 to 3.284) treated with platinum combination chemotherapy [43]. However, whether SNPs or haplotypes were associated with clinical outcome was still under debate since lacking of enough evidence.

To interpret the results of meta-analysis, several important acknowledgments should be addressed. First, did the *BRCA1* assessment methodology consistently? As we know, IHC detects gene expression at protein level, while RT-PCR assays at mRNA level. From mRNA to protein, many factors such as transcription, post-transcriptional regulation, translation and post-translation may affect this process. Besides, RT-PCR uses the bulk tumor/tissue to extract RNA, while IHC can distinguish cell type and can read protein level only in cancer cell when compared with normal epithelial cell. Even in studies using IHC or RT-RCR assessment methodology, their cutoff value was inconsistently. Although in subgroup analysis based on *BRCA1* detecting methods in platinum-based treatment, both IHC and RT-PCR showed the significant association between *BRCA1* level and ORR, the potential heterogeneity may exist. Also, what’s the proper cutoff that could predict the chemotherapy efficacy to a great extent? We are looking forward the future researches explore this relationship. Second, is the platinum-based chemotherapy the pure platinum and the toxal-based chemotherapy the pure toxal? *BRCA1* gene shows the different mechanism and efficacy in platinum and toxal regimens. As cell experiments suggest that low/negative *BRCA1* benefit from platinum whereas high/negative BRCA1 benefit more from anti-tubulin regimen such as paclitaxel and docetaxel. But in practice, single agent in chemotherapy is impossible as the limited efficacy. Platinum is usually combined with anti-tubulin agents, for example, toxal and platinum (TP), docetaxel and carboplatin (DC). In our meta-analysis, we sorted the studies into platinum-based studies means that every patient received platinum agents (cisplatin, carboplatin or oxaliplatin), the toxal-based chemotherapy means that every patient received toxal contained agents (toxal, taxane or docetaxel). Although our meta-analysis showed that patients with low/negative *BRCA1* have better objective response rate and longer OS and EFS, and patients with high/positive *BRCA1* have better ORR, the confounding factors from chemotherapy agents may exist in studies. Third, is *BRCA1* an important predict or prognosis factor to the clinical outcome? Many factors may contribute to the ORR, OS as well as EFS, for example, age, smoking status, pathological type, tumor stage, the drug dosage and treatment cycle, also the genetic as well as gene-environment interaction also involve in disease progression, there were not enough baseline characters that ensure us to conduct stratified analysis. Four, were all relevant studies included in the analysis? This is impossible and difficult to assess. Although we searched and collected relevant studies until the December 10, 2012 to our best effort, there were still some studies with negative results did not presented the sufficient data and some were failed to get published. In our meta-analysis, only 3 Caucasian studies including 197 patients evaluated the ORR in platinum-based treatment. In toxal-based chemotherapy studies, only 4 studies consisted of 376 patients evaluated this association. The small sample size may mislead us and draw a wrong conclusion. Besides, except for one multi-center study [31], our included samples were mainly distributed in some countries in East-Asian (Chinese and Japanese) and European (Spanish, Greece). So few studies could we found in other countries such us USA, Canada, UK, German, France and so on. Also, the African population was limited. This disequilibrium of population distribution may also affect our results.

## Conclusions

Despite the limitations of this meta-analysis, our study confirmed that low/negative *BRCA1* expression was associated with better objective response rate (ORR) and longer overall survival (OS) and event-free survival (EFS) in NSCLC patients treated with platinum-containing regimen, while high/positive *BRCA1* level were associated with better objective response rate in toxal contained regimen. Therefore, *BRCA1* might serve as a valuable marker for personal chemotherapy. However, considering the limitation our meta-analysis, multi-center of larger studies with hundreds or thousands of subjects and strict designed methodology was expected.

## Abbreviations

BRCA1: Breast cancer susceptibility gene 1; NSCLC: Non-small-cell lung cancer; OR: Odds ratio; ORR: Objective response rate; OS: Overall survival; EFS: Event-free survival; PFS: Progression-free survival; DFS: Disease-free survival; TTP: Time to progression; RT-PCR: Reverse-transcriptase polymerase chain reaction; IHC: Immunohistochemistry.

## Competing interest

The authors declare that they have no conflict of interest.

## Authors’ contributions

YYL and XL conceived and designed the study, YYL and XYL participated in selecting study, extracting data, performing the statistical analysis and drafting the manuscript. XL has been involved in revising the manuscript critically for important intellectual content. All authors read and approved the final manuscript.

## Funding

This reseach was supported by Guangxi Scientific reseach and technology development projects (Grant No.10124001A-44).
